# Evolution of Multidimensional Poverty in Crisis-Ridden Mozambique

**DOI:** 10.1007/s11205-022-02965-y

**Published:** 2023-03-14

**Authors:** Eva-Maria Egger, Vincenzo Salvucci, Finn Tarp

**Affiliations:** 1 UNU-WIDER, Helsinki, Finland; 2 University of Copenhagen, Copenhagen, Denmark

**Keywords:** First-order dominance, Mozambique, Multidimensional poverty, Multidimensional poverty index, I32

## Abstract

Mozambique experienced important reductions in the poverty rate until recently, before two major natural disasters hit, an armed insurgency stroke in the northern province of Cabo Delgado, and the country started suffering from a hidden debt crisis with associated economic slowdown. As the last available national household expenditure survey is from 2014/15, just before these crises started unfolding, there is need for a poverty assessment based on alternative data sources. We study the evolution of multidimensional poverty in Mozambique using survey data from the Demographic and Health Surveys (DHS). Using both the standard Alkire–Foster multidimensional poverty index and the first-order dominance (FOD) method, we find that the multidimensional poverty reduction trend observed between 2009–11 and 2015 halted between 2015 and 2018. Meanwhile, the number of poor people increased, mainly in rural areas and in the central provinces. Importantly, the poorest provinces did not improve their rankings over time, and between 2015 and 2018, no progress took place for most areas and provinces, as measured by the FOD approach.

## Introduction

Emerging from a devastating and prolonged armed conflict during the 1980s and early 1990s, Mozambique entered a period of sustained economic growth. Prior to 2014/15, the country managed to reduce both consumption and multidimensional poverty (see among others Arndt et al., 2012, 2015, 2016b, 2018; Brück & Van den Broeck, 2006; DEEF, 2016; DNEAP, 2010; DNPO, 1998, 2004; INE, 2004, 2010, 2015; Mahdi et al., 2018; Mahrt et al., 2020; World Bank, 2017). In international comparison, the gains registered by Mozambique over the 18-year span from 1996/97 to 2014/15 covered by the surveys in reference are notable. The consumption poverty headcount-ratio fell by about 25 percentage points and multidimensional poverty incidence by 21 percentage points. However, variations by areas/province persist, with multidimensional poverty being worse for the northern and central regions of the country and for rural areas (DEEF, 2016).

However, this is not an up-to-date picture as recent developments suggest. Following the years of favourable growth, various factors contributed to a severe economic downturn that started in 2015. They include a reduction in the prices of some of the most important exported goods (e.g., coal and gas) in combination with weaker international demand resulting from the economic crises in Europe, South Africa, and other key trading partners. A series of severe weather shocks also hit Mozambique after 2015, causing widespread damage and distress in various areas of the country.^1^ Furthermore, violent attacks by Islamist groups and unknown actors started occurring in the northern province of Cabo Delgado in late 2017. The attacks often targeted villages and thus created insecurity and displacement for the local population. Following the discovery of vast reserves of coal, minerals and natural gas in the 2010s, unrealistic revenue expectations from the extraction of natural resources became widespread. Moreover, the relationship with donors and international institutions started changing, and in 2015 a hidden debt crisis broke out (Arndt et al., 2007; De Renzio & Hanlon, 2007; Hanlon, 2017, Kroll, 2017; MNRC, 2017a, 2017b; Navarra & Udelsmann Rodrigues, 2018; Tvedten & Orre, 2016; Vollmer, 2013; World Bank, 2017). As a consequence, (i) the International Monetary Fund suspended its support to the country; and (ii) foreign aid and direct state budget support by development partners—which had already been on a downward trajectory—were further reduced and suspended, creating significant challenges for the management of public finances by drastically reducing the fiscal space (see World Bank, 2018).

These factor in combination led to a deep deceleration in the GDP growth rate, a first slowdown in 2015 and a second one, relatively bigger, in 2016 (see INE, 2017; World Bank, 2018). A rapid and significant depreciation of the national currency—the metical—followed with consequent increases in the prices of imported goods, causing an upsurge in domestic prices by around 40% between August 2014 and December 2016 (INE, 2017). This and the further reduction in foreign aid resulted in very limited fiscal space for price stabilization policies. Indeed, Mozambique is strongly dependent on imported goods, even those of first necessity (UNSD, 2017), including food. Moreover, the prices of food products, and especially basic food products, increased much more than the prices of non-food products. Mambo et al. (2018) analysed the consequences in terms of consumption poverty, suggesting a steep rise in consumption poverty due to the food price spike. There is, however, no up-to-date quantitative analysis from more recent years to assess the potential impacts of the economic crises on multidimensional poverty.^2^

A key question addressed here is the extent to which a flattening of the poverty reduction trend has occurred and whether poverty conditions might have worsened. We assess this question, which is by no means a trivial one, employing two competing, yet complementary methods to measure multidimensional poverty relying on representative household data covering the most recent period.

Mozambique is a relevant case study for several reasons. First, low frequency yet relatively good-quality data is available. Second, aid has supported the country’s development quite successfully (Macuane et al., 2017; Navarra & Udelsmann Rodrigues, 2018; The Economist, 2016). This is certainly so compared to elsewhere where the discovery of natural resources created high expectations while not delivering on socio-economic goals (Arndt et al., 2007; De Renzio & Hanlon, 2007; Hanlon, 2017; Tvedten & Orre, 2016; Vollmer, 2013). Third, the process of poverty reduction observed between 1996/97 and 2014/15 in Mozambique was quite notable, given the conditions of the country at the time of the Rome General Peace Accords that put an end to 17 years of war (Alden, 1995; DEEF, 2016).

Accordingly, we aim to estimate changes in multidimensional poverty during this recent crisis-ridden period, making use of up-to-date nationally representative household data, that is, the Demographic and Health Surveys/Malaria Indicator Survey 2018 (henceforth, DHS/MIS 2018) data. We compare the multidimensional poverty estimates emerging from the DHS/MIS 2018 with previous survey data for 2009, 2011 and 2015 belonging to the same DHS family. In contrast to Mambo et al. (2018), we measure the change in multidimensional poverty using actual data and employ as reference the DHS available from previous years. These data are not directly comparable to the 2014/15 Household Budget Survey data although some comparisons are feasible when due care is exercised.

Moreover, with respect to the multidimensional poverty measures, data issues regarding the individual indicators tend to be more straightforward than dealing with consumption data. Indeed, the indicators employed for multidimensional poverty analyses are relatively easy to observe. We therefore proceed to calculate a partial multidimensional poverty index (MPI) following Alkire and Foster (2011) as well as employing the more recent first-order dominance (FOD) method (Arndt et al., 2016a, 2016b). The latter does not impose a specific threshold to define households as poor. Instead, it uses multiple comparisons to assess which sub-population is better off than another one.

Given the discussion above, the contribution of our study is threefold. First, from the technical point of view, the application of the Alkire–Foster method is not novel per se. However, the comparison between this method and less-known methods like the FOD approach implemented here merits attention. It provides an important robustness check for the estimations presented and adds complementary insights on aspects that the Alkire–Foster approach cannot fully address (Aguilar & Sumner, 2020; Alkire et al., 2015b; Arndt et al., 2018; Fattore & Maggino, 2018; Kakwani & Silber, 2008; Permanyer & Hussain, 2018).

Second, from the empirical and policy point of view, our contribution is to the best of our knowledge the first attempt at measuring the trend in multidimensional poverty in Mozambique using nationally representative household survey data subsequent to the publication of the Fourth National Poverty Assessment in 2016 (DEEF, 2016). Until then, the government with support of its development partners designed and implemented a variety of anti-poverty policies. Following the International Monetary Fund’s (IMF) indications, Mozambique prepared its first poverty reduction strategy in the early 2000s, known as the PARPA, which contained and attempted to coordinate most of the anti-poverty policies and objectives of the country for the subsequent years. However, from 2014 on Mozambique has not had a specific strategic document for poverty eradication; all efforts of the country directed at fighting and reducing poverty started to be included in the operational matrix of the government’s five-year programme. This change in the way to tackle and monitor poverty in the country might have influenced the priority that the fight against poverty received, with less attention to anti-poverty policies after 2014. Thus, monitoring the process in poverty reduction with the most recent data available responds to this issue. Moreover, since 2014 a series of substantial shocks to poverty occurred. Mambo et al. (2018) analysed the consequences in terms of consumption poverty, suggesting a steep rise in consumption poverty due to the food price spike. We contribute a more comprehensive and updated assessment focussing on multidimensional poverty at national and sub-national level. While correlated with consumption poverty, multidimensional poverty captures longer-term development in various areas affecting people’s livelihoods. Furthermore, increases in consumption poverty can influence indicators that contribute to multidimensional poverty. For example, households tend to deplete assets that are easy to monetize to smoothen consumption in times of crisis (among others, see Baez et al., 2018; Dercon, 2005; Ellis et al., 2009; Groover et al., 2015; Lawson & Kasirye, 2013; Tschirley et al., 2006).

Third, and finally, our findings are relevant for other developing countries in the region and for countries that find themselves in similar conditions.

Our results suggest that poverty reduction did not only slow down during the 2015–18 period. In fact, both the methods adopted here reveal that the poverty reduction trend observed between 2009–11 and 2015 decelerated rapidly, and the poorest provinces have generally not improved their rankings over time. Moreover, the share of people with zero deprivations only slightly increased between 2015 and 2018, whereas the percentage of people with the maximum number of deprivations reduced modestly at national level, and it actually increased in urban areas, even if only slightly. In addition, the estimated probability of advancement between 2015 and 2018, as measured by the temporal FOD approach, is practically zero for most areas and provinces. These results point to a troubling intensification of overall deprivation when absolute numbers of people are considered. Due to sustained population growth, we estimate that the number of people, who are poor in a multidimensional sense, increased by approximately one million people in the period 2015–18, from about 21.3 to about 22.2 million people, mainly located in the rural areas of the central provinces.

Furthermore, we find that the changes in multidimensional poverty seem to be driven by changes in durable asset ownership. While housing or infrastructure, such as electricity or sanitation, are unlikely to disappear, it appears that households own fewer durable assets leading to higher deprivation scores. This aligns with the literature showing how in times of crisis assets are more frequently or more easily depleted to sustain consumption (among others, see Baez et al., 2018; Dercon, 2005; Ellis et al., 2009; Groover et al., 2015; Lawson & Kasirye, 2013; Tschirley et al., 2006). We do not claim to have established strict causality from the economic crisis to the poverty estimates. The complexity of the various factors contributing to the crisis and their variation in local or national impacts make that goal unrealistic at this point. However, establishing a set of updated poverty estimates does contain suggestive implications for the difficulties faced in promoting inclusive growth in Mozambique and the indisputable need to focus policy accordingly.

The paper proceeds as follows. We present the data in Sect. 2 and our methodology in Sect. 3; Sect. 4 contains the results and discusses them; while Sect. 5 concludes.

## Data

We use four sets of Mozambican data, all obtained from the DHS data repository. DHS data represent an outstanding resource in research, as all the surveys produced by the DHS Program are highly standardized and designed to be comparable.^3^ More specifically, we use: (i) DHS/AIDS Indicator Survey (AIS) 2009, (ii) DHS 2011, (iii) DHS/AIS 2015, and (iv) DHS/MIS 2018. The DHS was the original survey tool developed by The DHS Program, which recently created the AIDS Indicator Survey (AIS) and the Malaria Indicator Survey (MIS).^4^ As the denomination makes clear, these databases focus on demographic and health indicators: the DHS is more general, the DHS/AIS focuses on HIV/AIDS-related issues, whereas the DHS/MIS addresses malaria issues in detail. Depending on the specific survey, data have been collected and/or analysed by either the Ministry of Health (*Ministério da Saúde*, MISAU), the National Institute of Health (*Instituto Nacional de Saúde*, INS),^5^ the National Institute for Statistics (*Instituto Nacional de Estatística*, INE), or only by some of these institutions. Yet, ICF International supervised the work (see INS and ICF International 2019; MISAU et al., 2010, 2013, 2016).

With respect to the modules included in each of the surveys, DHS surveys are *“designed to collect data on marriage, fertility, mortality, family planning, reproductive health, child health, nutrition, and HIV/AIDS. Due to the subject matter of the survey, women of reproductive age (15–49) are the focus of the survey”* (DHS, 2021). However, a household questionnaire is always included, and some basic information is collected on the characteristics of each of the household members, including age, sex and education. On top, information on the characteristics of the dwelling, such as the source of water, type of sanitation facilities, materials used to construct the house, ownership of various consumer goods, is also reported (DHS, 2021).

Descriptive statistics for each of the four survey databases are in Table 1. From the above discussion, it is clear that even in those cases in which the focus is only on women or only on selected-age adults, all datasets are representative of households at the national and provincial levels, allowing for comparisons at these levels over time. In any case, the official reports produced based on the surveys used here clarify this point even further. In particular, the DHS/MIS 2018 report states that “*The Malaria Indicator Survey (IIM 2018) is a population-based survey, with national, urban and rural representation […]. The IIM 2018 sample design follows two phases that allow estimates […] for the following domains: national, urban and rural areas, each of the eleven provinces, namely Niassa, Cabo Delgado, Nampula, Zambézia, Tete, Manica, Sofala, Inhambane, Gaza, Maputo Province, and Maputo City*”^6^ (INS & ICF International, 2019).

**Table 1:** Basic information on the survey data used in the analysis. *Source*: Authors’ computation based on DHS (2020)

Survey	Year	Households sample	Male sample	Female sample	Fieldwork
DHS/AIS	2009	6097	All men	All women	June 2009–September 2009
			Age: 15–64 years	Age: 15–64 years	
			Sample size: 4799	Sample size: 6413	
DHS	2011	13,919	All men	All women	June 2011–November 2011
			Age: 15–64 years	Age: 15–49 years	
			Sample size: 4035	Sample size: 13,745	
DHS/AIS	2015	7169	All men	All women	May 2015–September 2015
			Age: 15–59 years	Age: 15–59 years	
			Sample size: 5283	Sample size: 7749	
DHS/MIS	2018	6196	No male respondents	All women	April 2018–June 2018
				Age: 15–49 years	
				Sample size: 6184	

From the discussion above, it follows that it is possible to analyze outcomes of interest measured at the levels for which the surveys are representative (national, urban/rural, provincial). This entails that, even though the coverage, the male–female balance and other characteristics of the surveys considered are different, and the individuals interviewed are not the same over time, it is still possible to compare, say, the proportion of households with access to safe water in a certain region over time. Based on the information provided in the official reports, we can then proceed to select several indicators of multidimensional poverty.

One limitation of the DHS/AIS/MIS surveys used in the present analysis is that the databases do not provide information on consumption, so we cannot use them to estimate the evolution of consumption poverty directly. Yet, they do contain a great deal of information on various indicators of multidimensional poverty. Therefore, we can use them to assess the evolution of multidimensional poverty over the period of interest.

## Methodology

Both analytical methods applied in this study form part of a stream of literature and analysis that has developed from at least the mid-1970s, which recognised poverty as a multidimensional phenomenon. They developed as a complement or alternative to more standard methods based on income or consumption and started to consider several non-monetary deprivation dimensions to assess poverty. Both the Alkire–Foster and the FOD methods are part of a broader category of analytical methods that compute multidimensional poverty based on the joint distribution of deprivations, thus requiring that the relevant information on each dimension of deprivation is available for each unit of analysis (Alkire et al., 2015b).

The multi-dimensional poverty literature and its associated ways of measurement have obvious roots back to the seminal capability approach developed by Sen (1974, 1979a, b, 1985 among others).^7^ Alkire (2016) stresses, when one passes from theory to the operationalisation of a policy-relevant measurement methodology, compromises are required. The data available for the measurement of capabilities represent a first constraint. Indeed, most data reflect achieved functionings rather than capabilities. Nonetheless, Alkire (2016) defends that it is possible to shed some light on capabilities even through a measurement methodology which is constrained to only use functionings information. Alkire et al. (2015a, 2015b, ch. 6), based on Alkire and Foster (2008), also argue that in some cases the Alkire–Foster outcomes, particularly the adjusted poverty incidence, could be interpreted as a measure of poverty related to capabilities.^8^

The methodology implemented here consists of four steps: (i) selection of the indicators for the multidimensional poverty analysis; (ii) analysis of the temporal trend of deprivation in each of the selected indicators; (iii) aggregation of the information into a Multi-dimensional Poverty Index (MPI), using the Alkire–Foster approach; and (iv) analysis of multidimensional poverty using an alternative methodology for multidimensional deprivation assessment, based on FOD. We describe each step in what follows.

First, we made the selection of indicators based on the existing literature and on the availability of well-being indicators in the four surveys. With respect to the literature, we mainly used as reference the dimensions and indicators found in the global MPI (UNDP and OPHI, 2019).^9^ However, not all the indicators were available in the surveys mentioned. In particular, the indicators included by the Oxford Poverty and Human Development Initiative (OPHI) in the dimensions of health and education were not available in most of the surveys considered. Consequently, we ended up with a shorter list of indicators, corresponding to the dimensions defined as “living standards” in the global MPI 2019. We thus selected cooking fuel, sanitation, drinking water, electricity, housing, and assets. Access to quality sanitation and to safe drinking water can also be classified as “health drivers”, “health determinants” or “health correlates”,^10^ but as we follow the global MPI approach, we have grouped them under the “living standards” dimension.

The living standards dimension is only one of the three dimensions considered in the global MPI, so it cannot capture the full complexity of multidimensional poverty. Yet, Alkire et al. (2020) estimated its contribution to overall poverty to be above 50%, with the contributions for health and education being 17.2 and 35.5%, respectively (Alkire et al., 2020).

The definitions for these indicators (see Table 2, which also includes weights, discussed further below) closely reflect the definitions in UNDP and OPHI (2019), with very small changes due to the unavailability of a few variables in some or all of the surveys considered. Regarding cooking fuel, a household is considered deprived if it cooks with dung, agricultural crop, shrubs, wood, charcoal, or coal. With respect to sanitation, the household is deprived if its sanitation facility is not improved [according to sustainable development goal (SDG) guidelines] or if it is improved but shared with other households.

**Table 2:** Dimensions, indicators, deprivation definitions, and weights. *Source*: Authors' adaptation from Alkire et al. (2019b)

Dimension of poverty	MPI indicator	Deprived if …	Weight
Living standards	Cooking fuel	A household cooks with dung, agricultural crop, shrubs, wood, charcoal, or coal	1/6
	Sanitation	A household’s sanitation facility is not improved (according to SDG guidelines) or it is improved but shared with other households	1/6
	Drinking water	A household does not have access to improved drinking water (according to SDG guidelines) or safe drinking water is at least a 30-min walk from home (as a roundtrip)	1/6
	Electricity	A household has no electricity	1/6
	Housing	A household has inadequate housing: the floor is made of natural materials or the roof or walls are made of rudimentary materials	1/6
	Assets	A household does not own more than one of these assets: radio, television, telephone, computer, animal cart, bicycle, motorbike, or refrigerator, and does not own a car or truck	1/6

MPI = multidimensional poverty index. A household is considered to have access to improved sanitation if it has some type of flush toilet or latrine, or ventilated improved pit or composting toilet, provided they are not shared. A household has access to clean drinking water if the water source is any of the following types: piped water, public tap, borehole or pump, protected well, protected spring or rainwater; and safe drinking water is at most a 30-min walk from home (as a roundtrip). A household is deprived in housing if the floor is made of mud/clay/earth, sand, or dung; or if the dwelling has no roof or walls or if either the roof or walls are constructed using natural materials such as cane, palm/trunks, sod/mud, dirt, grass/reeds, thatch, bamboo, sticks, or rudimentary materials such as carton, plastic/polythene sheeting, bamboo with mud/stone with mud, loosely packed stones, adobe not covered, raw/reused wood, plywood, cardboard, unburnt brick, or canvas/tent

Furthermore, a household is deprived with respect to drinking water if the household does not have access to improved drinking water (according to SDG guidelines) or safe drinking water is at least a 30-min walk from home (as a roundtrip)^11^; and if a household has no electricity, we also say it is deprived. Regarding housing, the household is considered as having inadequate housing if the floor is made of natural materials or the roof or walls are made of rudimentary materials. With respect to assets, we classify the household as deprived if it does not own more than one of the following assets: radio, television, telephone, computer, animal cart, bicycle, motorbike, or refrigerator, and does not own a car or truck.^12^

As noted, we apply two distinct methods for evaluating multidimensional poverty using the indicators identified. First, we apply the Alkire–Foster method for deriving an MPI (Alkire & Foster, 2011). This approach applies weights to a series of binary deprivation indicators where we divide the population into those considered deprived and those not deprived for each indicator. For example, in the analysis presented here, a household is deprived in its access to safe water if its source of drinking water is an unprotected well, a protected or unprotected spring, a river/dam/lake/pond/stream/canal or other unspecified sources. This indicator is given a weight of 1/6 (see last column in Table 2). Households deprived in indicators whose weights sum to a value greater than a cut-off (0.40; i.e., three or more out of the six selected indicators) fall in the multidimensional poor category.^13^ This multidimensional poverty headcount is then combined with a measure of distance below the cut-off to account for the fact that households deprived in dimensions summing to a weight of 0.40 are worse off than those summing to a weight of 0.20 are. The product of the headcount and the distance measure is the Alkire–Foster MPI. To be clear, there is no theoretical guidance on the weights and cut-offs applied. We chose equal weights for all indicators (1/6), and the 0.4 cut-off corresponds to being deprived in at least three out of the six indicators.^14^

Second, we apply a relatively recent method based on the concept of FOD.^15^ This approach relies on the proposition that not being deprived is better than being deprived. With multiple binary indicators, it is possible to identify states that are demonstrably better (i.e., not deprived in all dimensions) and states that are demonstrably worse (i.e. deprived in all dimensions). Using bootstrap methods, it is possible to derive a probability that a population is trending towards unambiguously better states. Our two methods rely on essentially the same data in complementary ways. The Alkire–Foster method has been widely used across Sub-Saharan Africa and beyond and is simple to apply; however, as noted, it requires an explicit, arbitrarily assigned weight associated with each dimension as well as assumptions regarding a cut-off point, which separates poor from non-poor households. The FOD approach has been less widely used and is somewhat less straightforward to apply/interpret; however, it does not require any assumptions with respect to the relative importance of the different dimensions of multidimensional poverty.

As Arndt et al. (2016a, 2016b: 6) put it, ‘the FOD criterion, in specific, corresponds to what in probability theory is referred to as the *usual* (*stochastic*) *order* (Lehmann, 1955)’. This implies that the FOD approach does not depend on arbitrarily applying a weighting scheme and cut-offs (Arndt et al., 2012). It simply assumes that not being deprived is better than being deprived for any considered dimension.

To illustrate the intuition behind FOD, let us suppose that we have data for five binary deprivation indicators on populations A and B, and we wish to determine whether population A is unambiguously better off than population B based on these indicators. The respective populations can be divided into 2^5^ = 32 different possible states corresponding to whether they are deprived or not deprived in the various indicators. Obviously, those not deprived in any indicator are best off and those deprived in all indicators are worst off. If we define 0 as deprived and 1 as not deprived, then the state (0, 1, 1, 0, 0) is unambiguously better than (0, 0, 1, 0, 0) because the former state is always at least equivalent and is better than the latter in one instance. However, the states (1, 0, 1, 0, 0) and (1, 1, 0, 0, 0) are indeterminate because each state is better than the other in one indicator, and the state (1, 1, 0, 1, 1) is not unambiguously better than the state (0, 0, 1, 0, 0) because no judgement is made as to the relative importance of indicator three versus all other indicators.

Formally, population A first-order dominates population B if one can generate the shares of the population in each state in population B by shifting probability mass within population A to states that are unambiguously worse (for a generalization of the methodology and a more formal presentation, see Arndt & Tarp, 2017; Arndt et al., 2012, 2016a, 2016b). Following Copeland (1951), complete welfare rankings of regions can be generated by, for example, counting the number of times a given region dominates other regions and subtracting the number of times the same region is dominated by other regions generating a score in the interval [− 99, 99]. Regions can then be naturally ranked with higher scores superior to lower scores and a Copeland index can be defined where all scores are normalized to fall in the interval [− 1, 1].

To help overcome the issue of indeterminate comparisons, suppose that neither A nor B dominates the other, and that on net A dominates 20 other regions, while B dominates negative one (i.e., the total number of regions that dominate B is one larger than the number of regions that B dominates). Then, it is sensible to rank A above B as in the Copeland index. Moreover, and importantly, it is also possible to use the FOD criterion to determine whether multidimensional poverty has unambiguously been improving through time. The comparison of each region with itself at a different point in time naturally yields only one comparison pair, but use of bootstrapping can help to mitigate the two disadvantages associated with the FOD approach through the generation of multiple comparisons (Arndt et al., 2012). Failure to advance through time implies that the distributional changes observed over time do not represent an unequivocal improvement over conditions that existed in the past. The FOD approach requires progress across all indicators and across the range of the welfare distribution, (i.e. also progress for the poorest is required; see Arndt & Tarp, 2017; Arndt et al., 2012, 2016a, 2016b).

It is important to highlight that consistency between the FOD and Alkire–Foster methods is not automatic. The FOD criteria are strict. While Alkire–Foster permits rapid progress in one indicator to overcome (or substitute for) declines in another indicator, the FOD does not. The same is true for population subgroups. With Alkire–Foster, rapid progress near the 0.40 cut-off point can overcome declines in multidimensional poverty for poorer groups. This is not the case for FOD. To register progress, FOD demands progress in all indicators and across all population subgroups (defined by the distribution of deprivations).

Results from the FOD analysis in DEEF (2016) showed that at the national level, the probability of advance is one (or 100%) for all period pairs considered with the notable exception of the 2002/03–2008/09 period where the probability of advance fell to 0.68. Due to the strict nature of the FOD criteria combined with the effects of sample size, probabilities of advance tend to decline when the data are disaggregated by zone or region (and the sample size is commensurately smaller). In terms of distribution of gains, the FOD approach focuses on whether or not there exists unambiguous improvement. In what follows, we will see that the results with respect to probability of advancement during recent years is inferior to what was found in DEEF (2016) for the period 2008/09–2014/15.

## Results

In this section, we present our main results regarding the temporal trends for each indicator (Sect. 4.1), the creation of the MPI following the Alkire–Foster approach (Sect. 4.2), and the multidimensional deprivation results obtained using the FOD methodology (Sect. 4.3). Several robustness checks are also performed and included in the “Appendix”. Lastly, we discuss results keeping in mind the relevant literature on the topic (Sect. 4.4).

### Descriptive Statistics

**Fig. 1:**
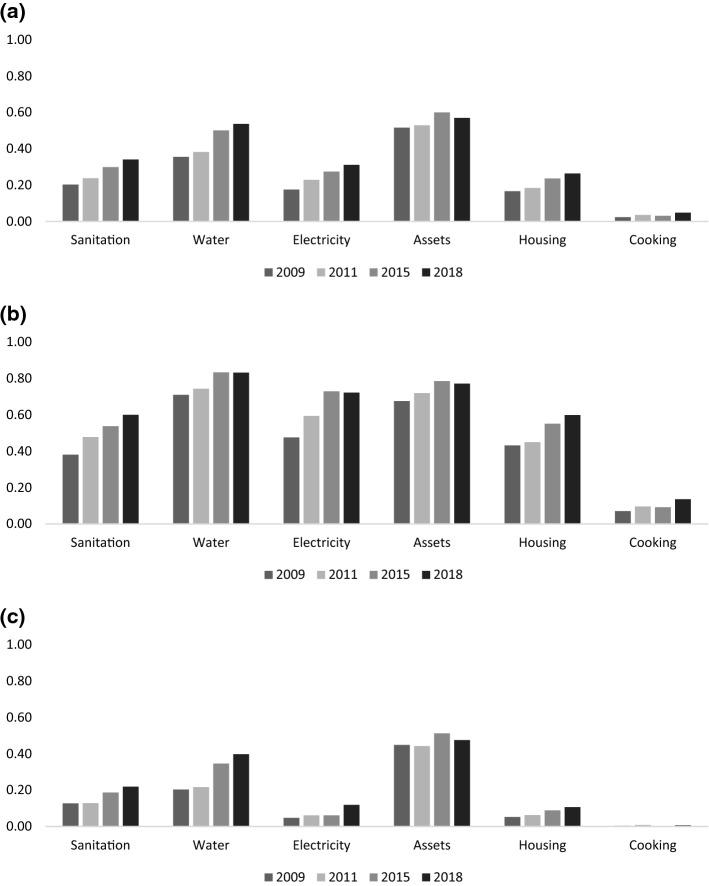
Proportion of non-deprived individuals for the selected welfare indicators: **a** national, **b** urban, and **c** rural levels, 2009–18. *Note*: Population weights are applied. *Source*: Authors’ computations

In Fig. 1, we introduce the proportion of individuals not deprived in each multidimensional poverty dimension and the underlying indicators. Some indicators had relatively low deprivation levels already in 2009 (drinking water, assets), whereas sanitation, electricity, housing, and especially cooking fuel presented much higher deprivation levels: the proportion of individuals not deprived is around 0.15 and 0.20 in 2009 and around 0.25 and 0.35 in 2018 for sanitation, electricity, and housing, whereas it is always below 0.05 for cooking fuel.

We observe improvement over time for most indicators. However, the trends vary among the indicators. Access to safe water increased notably between 2011 and 2015, but improved only slightly in subsequent years. With respect to sanitation, electricity, and housing, deprivation in these indicators steadily decreased between 2009 and 2018. The assets indicator shows a modest improvement between 2009 and 2011 followed by a significant increase in 2015 and a decrease in the proportion of individuals not deprived in this indicator in 2018. Even though the proportion of individuals owning a car, a motorbike, a refrigerator, or a mobile phone increased, there was a decrease in the proportion of individuals owning a bike or a radio, which explains the slight decrease in assets in 2018. With respect to cooking fuel, this is the indicator showing the highest levels of deprivation. While the proportion of individuals not deprived in cooking fuel doubled between 2009 and 2018, it did not exceed 0.05 at national level.

**Fig. 2:**
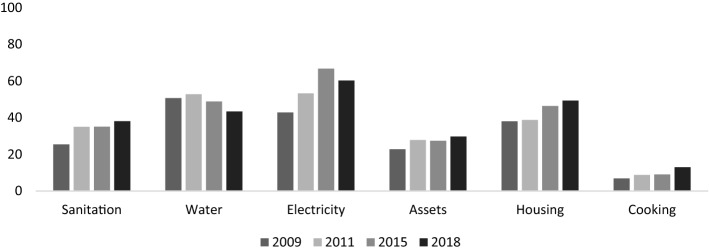
Urban–rural deprivation gap, percentage points, 2009–18. *Note*: Population weights applied. *Source*: Authors’ computations

Substantial differences also emerge when the deprivation indicators are broken down to urban and rural levels. In particular, rural households are on average more deprived than urban ones in all the indicators, and differences are sometimes substantial. Moreover, the deprivation gap between rural and urban areas increased over time for all indicators, except for access to safe water and for electricity in more recent years (Fig. 2). Even though urban areas are less deprived than rural ones, it is at urban level that we observe stagnating or slightly worsening conditions with respect to three out of six indicators in the period 2015–18 (water, electricity, and assets).

**Fig. 3:**
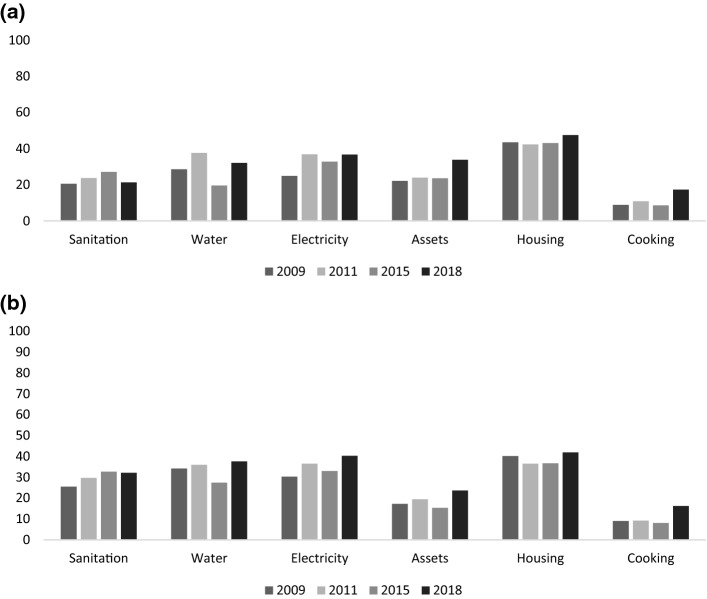
**a** South–north and **b** south–centre deprivation gaps, percentage points, 2009–18. *Note*: Population weights applied. *Source*: Authors’ computations

Conversely, rural areas experienced a non-negligible improvement in sanitation, water, and electricity, starting either in 2015 or 2018. At regional level, we also observe large differences in all welfare indicators between the northern and central regions and the south. In particular, the north and the centre show much higher levels of deprivation in all indicators and in all years. Excluding sanitation, for all the other welfare indicators the gap between the south and the other two regions increases in the period 2015–18. We present urban–rural gaps and south–north and south–centre gaps in Figs. 2 and 3, respectively.

**Fig. 4:**
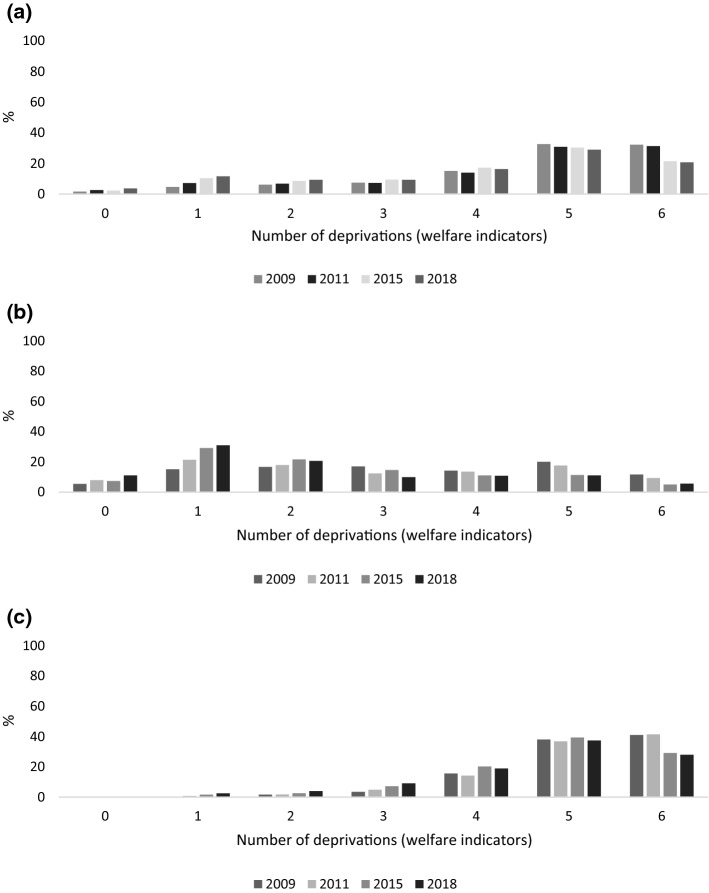
Percentage of individuals deprived in 0, 1, 2, 3, 4, 5, or 6 welfare indicators, 2009–18: **a** national, **b** urban, and **c** rural samples. *Note*: Population weights applied. *Source*: Authors’ computations

It is also interesting to show the percentage of individuals deprived in 0, 1, …, 6 indicators. Figure 4 shows this for the entire country and for rural and urban areas.

First, it emerges that at national level, and especially in rural areas, the percentage of individuals who suffer no deprivation in the indicators selected is very limited. This percentage slightly increased over time, but it did not exceed 4% at national level and it remained close to zero for rural areas. Conversely, the percentage of individuals deprived in only one of the six indicators steadily increased over time, from about 5 to 12% over the period considered. On the other hand, the share of individuals deprived in five out of six deprivation indicators gradually decreased from 2009 to 2018, from about 33% to a level of about 29%.

The share of individuals deprived in all the indicators is also a very important indicator of advancement and it shows a slight decrease between 2009 and 2011, a sharp drop between 2011 and 2015, and stagnation afterwards. Looking more thoroughly at urban–rural differences, we find that the share of individuals deprived in five out of six indicators in rural areas is mostly constant over time and high in absolute levels (slightly less than 40% of the rural population). Instead, the percentage of individuals deprived in all indicators dropped between 2011 and 2015, but then remained mostly constant (from 29 to 28%). In contrast, the improvement observed in urban areas with respect to households deprived in just one of the indicators is impressive (from about 15–31% in 9 years). This is also reflected in the decreasing percentage of individuals deprived in five and six indicators, which is clearly observed up to 2015; conversely, in 2018, we notice a stagnation or reversal in both percentages.

### Alkire–Foster Results

We proceed to create an MPI using the Alkire–Foster approach. In particular, we first apply weights to our binary indicators (sanitation, water, electricity, assets, housing, and cooking fuel). Next, we establish a cut-off and households deprived in indicators whose weight sums to a value greater than the cut-off are considered poor. Finally, this multidimensional poverty headcount, or poverty incidence, indicated with *H*, is combined with a measure of distance below the cut-off, the poverty intensity, *A*, to create the MPI, *M*^*0*^ (for details, see Alkire & Foster, 2011; Alkire et al., 2015a, 2015b).

In this analysis, we assign a weight of 1/6 to each of the six indicators selected. This is in line with the global MPI (UNDP and OPHI, 2019) that assigns the same weight to all the indicators contained in each individual dimension. Given that we only consider the dimension defined as ‘living standards’ in the global MPI, each indicator is assigned a weight of 1/6 (Table 2). The cut-off is as already explained set at 0.40 in the baseline analysis. A sensitivity analysis is subsequently performed with different cut-offs. The results relative to poverty incidence, *H*, poverty intensity, *A*, and the MPI, *M*^*0*^, are presented for the years 2009, 2011, 2015, and 2018 in Table 3, for the entire country, at rural–urban and regional levels.

**Table 3:** Poverty incidence, *H*, poverty intensity, *A*, and MPI, *M*^*0*^, national, urban–rural, and regional levels, 2009–18. *Source*: Authors’ computations

Level	Year	*H*	*A*	*M*^0^	Observations
National	2009	0.874	0.837	0.732	25,752
	2011	0.833	0.839	0.699	61,842
	2015	0.786	0.802	0.63	32,550
	2018	0.754	0.802	0.605	28,723
Urban	2009	0.627	0.736	0.461	11,608
	2011	0.527	0.742	0.391	23,632
	2015	0.419	0.693	0.291	14,624
	2018	0.374	0.721	0.27	12,109
Rural	2009	0.98	0.864	0.847	14,144
	2011	0.973	0.863	0.84	38,210
	2015	0.958	0.824	0.79	17,926
	2018	0.933	0.817	0.762	16,614
North	2009	0.939	0.846	0.794	7,128
	2011	0.922	0.855	0.789	15,464
	2015	0.891	0.804	0.717	9,259
	2018	0.861	0.805	0.693	7,361
Centre	2009	0.942	0.857	0.807	9,796
	2011	0.915	0.848	0.776	23,815
	2015	0.862	0.821	0.708	11,493
	2018	0.851	0.819	0.697	10,920
South	2009	0.647	0.763	0.493	8,828
	2011	0.557	0.776	0.433	22,563
	2015	0.527	0.75	0.395	11,798
	2018	0.423	0.73	0.309	10,442

Population weights applied

Since the poverty intensity stayed broadly constant over the period considered, the trend of MPI closely reflects what happened to poverty incidence. In general, multidimensional poverty levels remained high in Mozambique, even though a gradual improvement is noticeable. As for the trend observed for some of the underlying indicators, the reduction in the MPI levels is more pronounced between 2009 and 2011 and between 2011 and 2015 than during 2015–18. The multidimensional poverty incidence (*H*) is found to be significantly different between 2009 and 2011 and between 2015 and 2011, but the difference is not statistically significant between 2015 and 2018. With respect to the MPI, *M*^*0*^, only the difference between 2015 and 2011 is statistically significant, whereas the differences between 2011 and 2009 and between 2018 and 2015 are not statistically significant.^16^

**Fig. 5:**
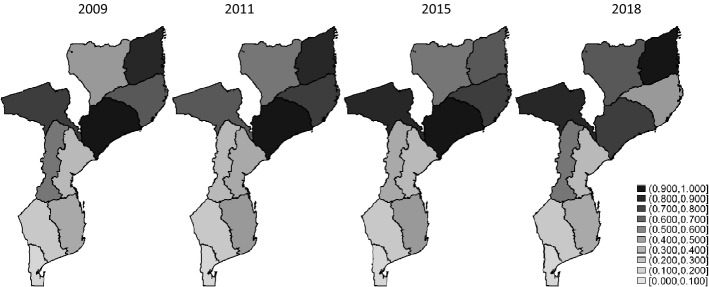
MPI, *M*^*0*^, at provincial level, 2009–18. *Notes*: Population weights applied. In the figure key, brackets and parentheses represent closed and open intervals, respectively. Accordingly, [0.000, 0.100] includes both 0.000 and 0.100, while (0.100, 0.200] does not include 0.100. It only comprises numbers greater than 0.100, including 0.200 and so on. *Source*: Authors’ computations

According to the multidimensional poverty results, computed using the Alkire–Foster method, the gap between urban and rural areas is wide and increasing over time. Furthermore, the gap between the southern region and the rest of the country is significant, with respect to both poverty incidence and the MPI. At the provincial level, we observe from Fig. 5 that most provinces improved their situation with respect to MPI. However, it is also clear that the poorest provinces did not change their rankings much over time, so that the poorest provinces are still those located in the centre–north, with MPI values substantially higher than provinces in the south.

**Fig. 6:**
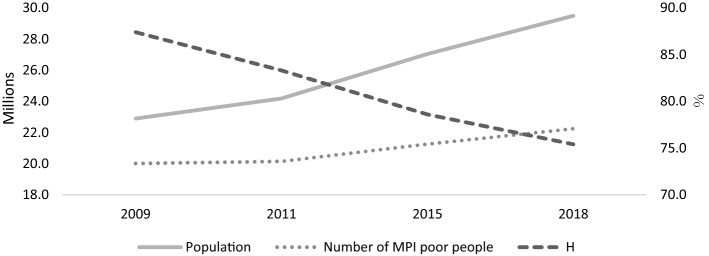
Multidimensional poverty incidence (*H*), population, and number of multidimensional poor people (in millions), 2009–18. *Notes*: Population and number of MPI poor people shown on the left axis (millions), the multidimensional poverty incidence, *H*, on the right axis (%). Population weights applied. *Source*: Authors’ computations

Given the above-mentioned multidimensional poverty results, we can also compute the number of multidimensional poor people by multiplying the population in each given year^17^ by the poverty incidence; *H*.^18^ Results are in Fig. 6. The absolute number of multidimensional poor people remained constant between 2009 and 2011 (about 20 million individuals), but it increased afterwards. It reached about 21 million people in 2015, notwithstanding the big improvement observed between 2011 and 2015 in several welfare indicators, and it further went up to 22.2 million people in 2018.

The number of multidimensional poor people increased by approximately one million people in the period 2015–18, mainly located in the rural areas of the central provinces. Indeed, we estimate that in the same period the number of poor people in urban areas reduced by about 93,000 people and the number of poor people in the southern provinces reduced by about 770,000 people. This reflects the fast population growth experienced by the country in recent years, but it also shows the kind of challenges Mozambique is facing when trying to reduce poverty in its various dimensions. Generating modest or even big improvements with respect to a few welfare indicators does not ensure that the number of multidimensional poor people decreases; more so in crisis-ridden times like the ones studied in this analysis. These results certainly point to a troubling intensification of poverty.

### FOD Results

We now turn to our main results obtained using the FOD approach involving the six welfare indicators previously selected. As explained in Sect. 3, population A first-order dominates population B if one can generate the shares of the population in each state in population B by shifting probability mass within population A to states that are unambiguously worse (Arndt & Tarp, 2017; Arndt et al., 2012, 2016a, 2016b). We can then count the number of times a given region dominates other regions (spatial FOD) and subtract the number of times the same region is dominated by other regions (net dominance), and normalize all scores to fall in the interval [− 1, 1]. This is indicated in the following tables as the probability of net dominance (i.e., the probability that a population dominates all other populations less the probability that a population is dominated by all other populations), interpreted as the cardinal measure of multidimensional well-being. We use the term ‘well-being’ here instead of multidimensional poverty because the measure intuitively captures positive outcomes and not deprivation as in the MPI. This provides the basis to rank populations (see Arndt & Tarp, 2017). We display the latter index for different areas of Mozambique in Table 4 and derive regional ranks as well. Comparisons include all provinces, urban and rural areas as a whole, and the national level.

**Table 4:** Spatial FOD multidimensional poverty comparisons, net dominance probabilities, and rankings of deprivation, 2009–18. *Source*: Authors’ computations

Area	Probability of net domination 2009	Ranking 2009	Probability of net domination 2011	Ranking 2011	Probability of net domination 2015	Ranking 2015	Probability of net domination 2018	Ranking 2018	Change in ranking 2009–18
Maputo City	1.000	1	1.000	1	1.000	1	0.990	1	0
Maputo Province	0.704	3	0.804	2	0.773	2	0.811	2	− 1
Urban	0.730	2	0.735	3	0.747	3	0.708	3	1
Gaza	0.045	4	0.060	4	0.150	4	0.190	4	0
Sofala	− 0.082	6	0.046	5	0.012	6	0.039	5	− 1
National	0.012	5	0.041	6	0.029	5	0.003	6	1
Inhambane	− 0.149	8	− 0.155	10	− 0.122	7	− 0.087	7	− 1
Nampula	− 0.197	9	− 0.328	11	− 0.280	10	− 0.248	8	− 1
Manica	− 0.234	10	− 0.025	7	− 0.247	9	− 0.252	9	− 1
Niassa	− 0.106	7	− 0.135	9	− 0.318	11	− 0.291	10	3
Tete	− 0.254	11	− 0.119	8	− 0.449	12	− 0.449	11	0
Cabo Delgado	− 0.541	14	− 0.614	13	− 0.242	8	− 0.452	12	− 2
Zambézia	− 0.498	13	− 0.790	14	− 0.545	14	− 0.477	13	0
Rural	− 0.429	12	− 0.519	12	− 0.507	13	− 0.485	14	2

Unsurprisingly, the capital city of Maputo dominates all other regions, followed by the Province of Maputo, the urban areas as a whole, and the southern province of Gaza. These four areas appear in the first four positions in all surveys considered. At the other end lie the northern–central provinces of Nampula, Manica, Niassa, Tete, Cabo Delgado, the rural areas as a whole, and Zambézia. The change in rankings is minimal. In the last column, we show the change in ranking between 2009 and 2018, and, excluding Niassa, other provinces did not move (either up or down) by more than two positions.

It is possible as well to use the FOD criterion to determine whether multidimensional well-being has been improving through time in the same area/region (temporal FOD), using bootstrapping to mitigate the fact that the comparison of each region with itself at a different point in time naturally yields only one comparison pair. The results are again normalized to fall in the interval [− 1, 1] and are presented in Table 5. The probabilities of advancement are larger on average when we compare 2015 and 2018 with 2009 and when we compare 2018 with 2011. Regional differences exist, but lower probabilities of advancement emerge when we compare 2015 with 2011 and 2011 with 2009. However, the lowest probabilities of advancement clearly appear when we contrast 2018 with 2015. In this case, most probabilities are around zero, the only ones respectively above or below 10% being Maputo Province, with a probability of 22%; the urban areas as a whole, with a probability of 11%; and the province of Cabo Delgado, showing a sizeable negative probability of − 15%.

**Table 5:** Temporal net FOD multidimensional poverty comparisons, 2009–18. *Source*: Authors’ computations

Area	2011 FOD 2009	2015 FOD 2009	2015 FOD 2011	2018 FOD 2009	2018 FOD 2011	2018 FOD 2015
National	0.44	0.95	0.17	1	0.99	0.03
Rural	0.07	0.08		0.7	0.22	0.04
Urban	0.47	0.89	0.29	0.99	0.84	0.11
Cabo Delgado	0.06	0.8	0.4	0.28	0.01	− 0.15
Gaza		0.43	0.46	0.6	0.52	0.05
Inhambane	0.01	0.14	0.04	0.31	0.4	0.01
Manica	0.37	0.06	− 0.02	0.14	− 0.01	0.02
Maputo City	0.01	0.13	0.08	0.63	0.31	
Maputo Province	0.26	0.71	0.12	0.94	0.89	0.22
Nampula	− 0.02	0.17	0.14	0.16	0.49	0.09
Niassa	0.01	0.04		0.02		
Sofala	0.03	0.48	0.12	0.7	0.17	0.06
Tete	0.01	0.03				
Zambézia	− 0.05	0.6	0.71	0.16	0.77	0.03

Empty cells indicate that the comparison is indeterminate, which entails that the results provide no evidence of improvement for some year in some area/region. Net probabilities of temporal FOD are obtained via bootstrap

The Cabo Delgado result likely reflects the ongoing insurgency in the region and possibly with some of the natural shocks experienced in the area. The results provide no evidence of improvement for some years in some areas/regions, as indicated by the blank cells in Table 5. Notably, there is no evidence of progress for the city of Maputo, for Niassa, and even for the coal-rich province of Tete between 2015 and 2018, and very little evidence of improvement at national level and for rural areas in the same period.^19^ The lack of advancement is likely due to the declines in assets at national level and in asset and other indicators at urban/rural and regional level, as evidenced in Fig. 1.

#### Robustness Checks

**Fig. 7:**
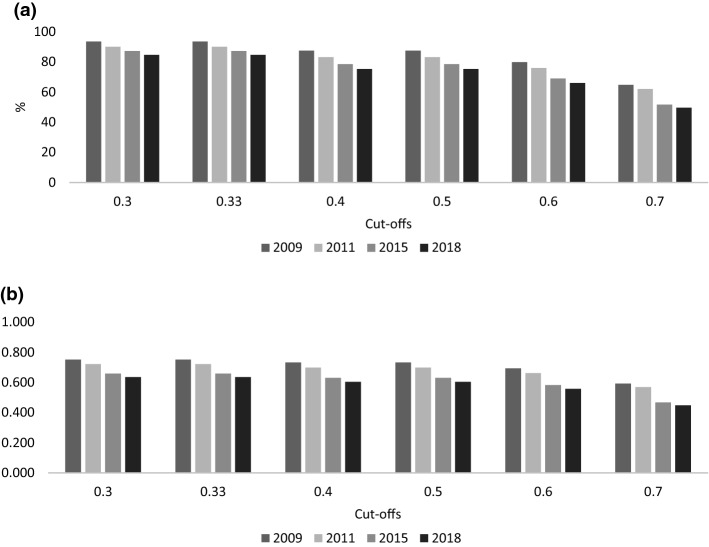
**a** Poverty incidence, *H*, and **b** MPI, *M*^*0*^, with different cut-offs, 0.30–0.70, 2009–18. *Notes*: Poverty incidence, *H*, as a percentage of the population, and MPI, *M*^*0*^, with different cut-offs: 0.30, 0.33, 0.40, 0.50, 0.60, and 0.70. Population weights applied. The difference between the poverty incidence, *H*, in 2015 and 2018 and the MPI, *M*^*0*^, in 2015 and 2018 is not statistically significant in any of the cases considered. *Source*: Authors’ computations

We performed a series of robustness checks with respect to both methodologies implemented, MPI and FOD. Full results are in the “Appendix”. Two concerns arise: the cut-off chosen in the MPI method and difficult indicators in both methods. If a large share of the population is concentrated around a certain level of deprivation, a higher or lower cut-off might lead to very different results. Some indicators might drive the results because very few people appear to be not deprived with respect to that indicator (this is the case of cooking fuel), or because the indicator shows a worsening trend between 2015 and 2018, as in the case of assets. We apply various cut-offs to the MPI and our main results in terms of the general trend observed in both the poverty incidence and the MPI persist (Fig. 7 in the “Appendix”). Regarding concerns of the influence of specific indicators, we implemented five alternative specifications that involve either adjusting the weights of indicators or dropping an indicator, or both. Again, our main results prevail (Table 6).

**Fig. 8:**
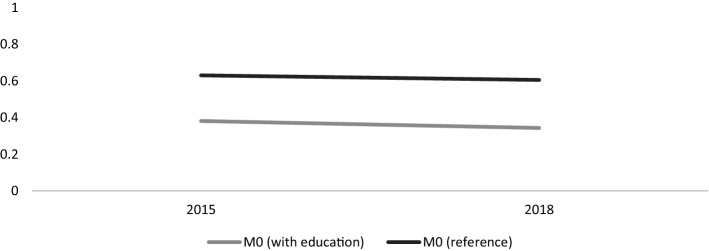
MPI, *M*^*0*^, original specification and including one indicator linked to education, 2015–18. The MPI including the education indicator, *M*^*0*^ (with education), is obtained using the following weights: years of education of the woman interviewed (1/2), Living standards (1/2) (which entails that: Sanitation = 1/12, Drinking water = 1/12, Electricity = 1/12, Assets = 1/12, Housing = 1/12, Cooking fuel = 1/12). *Source*: Authors’ computations

Lastly, in the FOD robustness analysis we drop both individual and several indicators. The results of spatial dominance do not change, but the temporal results do. Eliminating the asset indicator results in poverty reduction instead of stagnation. This aligns with our argument that assets might have played an important role during the crisis-ridden period in buffering against shocks (Tables 7 and 8 in the “Appendix”). Finally, we also perform a robustness check including one indicator linked to education, i.e. the years of education of the woman interviewed. This is not an indicator listed among those used in the standard MPI or in the Global MPI, so we did not consider it in our main estimations, but it may still provide an approximation of the education level in the household. Depending on the weight allocated to the latter indicator, the level of multidimensional poverty changes. However, the trends are confirmed and support the general results of the study (Table 9 and Fig. 8).

### Discussion of the Results and Further Steps

Looking at our results, we make a number of observations. Some of the results obtained in Sects. 4.1–4.3 are in line with multidimensional poverty results reported for Mozambique and other African countries (Alkire & Housseini, 2014; ; Alkire et al., 2017; Nissanke & Ndulo, 2017; Mahrt et al., 2020). Indeed, Alkire and Housseini (2014) show that the overall trend in multidimensional poverty in Africa has been declining, notwithstanding large variations across countries. Also, these authors argue that in some cases a decline in the national MPI can be due to a strong decline concentrated in only a few areas of the country under analysis, as for example in Nigeria, where the overall MPI declined, but the decline was concentrated in just one region where only 13% of the poor lived.

In the case of Mozambique, we also observe a large decline in the MPI in some regions, while other regions continue to lag behind. Alkire and Housseini (2014) and Nissanke and Ndulo (2017) also report that in sub-Saharan Africa rural areas show higher levels and intensity of poverty. We confirm these findings for Mozambique. Moreover, in their analysis of multidimensional poverty of children in Mozambique using the Alkire–Foster method, Mahrt et al. (2020) find that about half of the children in Mozambique could be considered multidimensional poor in 2014/15, with a substantial divide between urban and rural areas and between northern and southern provinces.^20^ Our findings confirm this divide in different data and for adults and children.

With respect to changes in the MPI, Alkire et al. (2017) present results on the intertemporal changes in the MPI and inclusiveness of multidimensional poverty reduction for 34 countries. They include Mozambique, using the 2003 and 2011 DHS data, and confirm a statistically significant decrease in the MPI at national and sub-national level. However, they do not go beyond 2011. When new data on education and health become available,^21^ we can establish whether the findings of a halt in the decreasing trend in the MPI reported in this analysis are confirmed or not using the full list of indicators implemented in the Global MPI and in most other papers based on the same approach.

The broad agreement in the results from the Alkire–Foster and FOD methodologies is an encouraging finding. Permanyer and Hussain (2018), for example, compared the performance of MPI and FOD with data from 48 DHS surveys from developing countries and concluded that the FOD approach could be implemented as a useful robustness check for poverty indices like the MPI; in particular, they find that the simultaneous application of the FOD methodology helps to distinguish those cases in which the comparisons are sensitive to alternative specifications from those which are not, and propose to use the FOD as a complement to the MPI, with the advantage that the former does not rely on many assumptions with respect to the construction of the index. Indeed, our robustness checks from Sect. 4.3.1 and from the “Appendix” reinforce this conclusion.

The two methodologies, MPI and FOD, have thus been used with some interesting results in different settings, including Mozambique (Arndt & Tarp, 2017; Arndt et al., 2016a, 2018; DEEF, 2016; Permanyer & Hussain, 2018). In particular, previous applications of the FOD methodology in Mozambique, performed before 2018, found first that the country registered fewer gains through time compared to other countries. Second, there are positive but sometimes very small probabilities of advance at most geographical levels (national, urban/rural, regions and provinces). Third, the relatively well-off regions are the urban zones, Maputo Province and Maputo City, while the relatively disfavoured areas include rural zones, Tete, Zambezia, Nampula, Niassa and Cabo Delgado (Arndt et al., 2012, 2016a; DEEF, 2016). As noted, some of these findings were confirmed in our analysis, whereas others seem to be specific to the period studied.

The research contained in this study has the potential of being expanded and/or developed in many ways. We identify at least three promising directions. First, Mozambique and other developing countries are increasingly producing higher frequency data, such as Computer-Assisted Telephone Interviewing (CATI) surveys (see Alkire, 2014; Croke et al., 2012). CATI surveys, which have already been implemented in Mozambique with some success concerning the transition from university to work or from vocational training to work (Jones et al., 2018, 2020), represent extremely interesting sources of data especially for what concerns multidimensional poverty evaluation and monitoring. Indeed, the indicators used in this kind of analyses are often easily observable and described by the respondents, particularly with respect to the living standards and, to a lesser extent, the education dimensions.

Second, more spatial data, remote sensing and geographical information are being made available, also for Mozambique and developing countries in general, which has been shown to represent a tool with great potential in the measurement of multidimensional poverty and poverty-related outcomes (Duque et al., 2015; Imran et al., 2014; Li et al., 2020; Liu & Xu, 2016; Pan & Hu, 2018; Shi et al., 2020; Sohnesen et al., 2020; Steele et al., 2017; Watmough et al., 2019). In the context of Mozambique, this could greatly help in the assessment of both poverty and well-being in remote areas such as the northern part of Cabo Delgado, where the armed insurgency prevents standard data collection. Another option in this category is more frequent assessment of poverty between major surveys in countries such as Mozambique, where household budget surveys are only collected every five or six years and where other kinds of surveys, such as the DHS surveys implemented in the present analysis, are also infrequent. Finally, these data are increasingly helpful in assessing the impact of natural disasters or policies on indicators relevant for (multidimensional) poverty (see Fisker et al., 2019; Malmgren-Hansen et al., 2020 for studies in this research area and using Mozambican data).

A third possibility to expand on the analysis contained in this study is represented by the application of the Alkire–Foster and FOD methods to the forthcoming comprehensive DHS data in Mozambique, (DHS, 2020). This database will permit to update the Global MPI for Mozambique, currently based on 2011 DHS data,^22^ as well as to evaluate multidimensional poverty changes after the Covid-19 crisis.

## Conclusions

Using the most recently available household survey data in Mozambique, we asked whether and how poverty has changed in a period of socio-economic crises and natural shocks. Employing two methods of multidimensional poverty measurement, the Alkire–Foster MPI and the FOD method, we found that the poverty reduction experienced up to 2015 slowed down significantly in the crisis-ridden period after 2015. In terms of the MPI, we noticed a statistically significant reduction of 0.07 percentage points between 2011 and 2015 in contrast to a non-statistically significant reduction of less than 0.03 points from 2015 to 2018. While the MPI is much higher in rural than in urban areas, this pattern of change over time is the same and the difference between 2015 and 2018 is not statistically significant in both areas.

Moreover, the number of multidimensionally poor people increased by approximately one million people in the period 2015–2018, from about 21.3 to about 22.2 million people. This points to an intensification of poverty, especially because most of the additional poor people are located in the already vulnerable rural areas and in the central provinces.

We also found that poverty intensity, meaning the share of households living with relatively more deprivations, remained constant during the crisis period and it increased in urban areas. The regional differences in poverty reduction are comparable to those in past poverty assessments. The poorest provinces have remained the same over time. The FOD analysis and the percentage net deprivation at regional level confirm this. The FOD analysis further reveals that the percentage of people with zero deprivation remained practically the same between 2015 and 2018—the difference is not statistically significant—and the same occurred with the percentage of people with six deprivations. The likelihood of an improvement in multidimensional deprivation in that period is practically zero.

We conclude that overall improvements in access to basic services, asset ownership, and housing conditions seem to have stalled in recent years explaining why we do not see a large increase in the share of households in the non-deprived category. At the same time, a large share of the population even lost some of their assets increasing their deprivation, which drives the rise in poverty intensity. The data show that this intensification is primarily due to an increase in households with asset deprivation. In contrast to housing characteristics and access to water, electricity, and sanitation, assets can be sold in times of dire need (among others, see Baez et al., 2018; Dercon, 2005; Ellis et al., 2009; Groover et al., 2015; Lawson & Kasirye, 2013; Newman & Tarp, 2020; Tschirley et al., 2006). Whether this helped the affected households to maintain their consumption levels during the crisis will only be possible to assess when an up-to-date household consumption survey is in hand.

Finally, although we cannot claim to have established causal linkages in this study, it is very likely that our results reflect the impacts of negative shocks during the 2015–18 period: economic crisis, hidden debt, natural disasters, and armed attacks in Cabo Delgado. The upcoming DHS data and the 5th national poverty assessment will be able to shed more light on the dynamics involved and whether Mozambique is returning to its path of inclusive growth.

The findings point at two conclusions for policy. One, the structural differences between rural and urban areas as well as the greater regions of the country must be addressed explicitly as they have continued to persist over decades. Expansion of infrastructure and basic services to the more deprived areas deserves prioritization. Two, the increase in asset deprivation indicates a lack of social protection in times of shocks. The government is already considering initiatives such as disaster insurance and expansions of basic social protection programs with relevant donors. This study provides evidence that this is indeed both highly needed and should be addressed as a policy priority.

## Figures and Tables

**Table 6:**
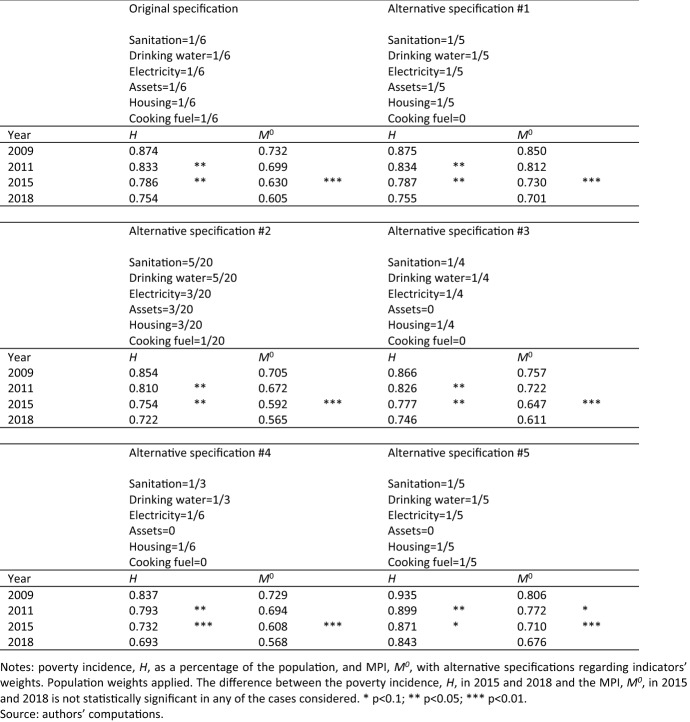
Poverty incidence, *H*, and MPI, *M*^*0*^, with alternative specifications regarding indicators’ weights, 2009–18. *Source*: Authors’ computations

Poverty incidence, *H*, as a percentage of the population, and MPI, *M*^*0*^, with alternative specifications regarding indicators’ weights. Population weights applied. The difference between the poverty incidence, *H*, in 2015 and 2018 and the MPI, *M*^*0*^, in 2015 and 2018 is not statistically significant in any of the cases considered

*p < 0.1; **p < 0.05; ***p < 0.01

## Appendix

In Sect. 4.3.1, we mentioned the results of the robustness checks performed in the present analysis. In this “Appendix” we present the robustness checks in more detail, with respect to both the methodologies implemented, MPI and FOD.

We start with the MPI and argue that changing the specification does not affect the main results. In particular, we show that when we modify the cut-off or change the indicator weights, this does not influence the results and their interpretation.

With respect to changes in the cut-off, Fig. 7 shows that the general trend observed in both the poverty incidence and the MPI with cut-off at 0.40 is not greatly affected by changes in the level of the cut-off. With higher cut-offs, the only noticeable differences are that the proportion of people considered poor decreases, the MPI levels are lower, and the difference between the poverty incidence and the MPI in 2015 and 2018 reduces. However, the difference between the poverty incidence in 2015 and 2018 and the MPI in 2015 and 2018 is not statistically significant in any of the cases considered (cut-off at 0.30, 0.33, 0.40, 0.50, 0.60 and 0.70).

Regarding the change in the specification of the indicators’ weights, we implemented five alternative specifications that seem to encompass most of the reasonable changes that one could be willing to propose given the data in hand and show the outcome in Table 6. In the alternative specification #1, we drop from the list of indicators the cooking fuel one, given that it might be that our results could have been influenced by the presence of an indicator for which most of the population is deprived. However, we show that this does not seem to be the case, as the difference between the poverty incidence in 2015 and 2018 and the MPI in 2015 and 2018 is not statistically significant in this case either. Nor is it significant in the subsequent alternative specifications. In the alternative specification #2, we assign a greater weight to those deprivation indicators that seem to be more important in the Mozambican case, i.e., water and sanitation. We assign a slightly lower weight to electricity, assets and housing, and give a very low weight to the cooking fuel indicator, for the reasons outlined.

In the alternative specification #3, we also get rid of the asset indicator, since this is the only indicator for which we observe a worsening trend between 2015 and 2018, which might be driving our results towards the outcome observed in previous sections. We stress this is not a particularly relevant/realistic case given the important role played by assets especially in a downturn. However, even eliminating the asset indicator does not change results with respect to the statistical significance of the difference between the poverty incidence in 2015 and 2018 and the MPI in 2015 and 2018. If we only maintain in our analysis water, sanitation, electricity and housing, but assign a higher weight to the first two indicators (alternative specification #4) results are not changed either. If we include back the cooking fuel indicator, and only leave aside the asset indicator, results do not change in this case as well. We also report the results for the original specification in the first panel to facilitate comparison of the various specifications with the one implemented in the main analysis.

Turning to the FOD, we cannot change the weights, because as explained the FOD methodology does not assign weights to the different indicators. Nonetheless, we can drop in this case as well the indicators that might be seen as “problematic”, either because very few people appear to be not deprived with respect to that indicator (this is the case of cooking fuel), or because the indicator shows a worsening trend between 2015 and 2018, as in the case of assets, which might be biasing the results.

In this case as well we report the original specification, then an alternative specification #1 in which the cooking fuel indicator is dropped, an alternative specification #2 in which the asset indicator is not considered, and an alternative specification #3 in which both the cooking fuel and the asset indicators are dropped.

Only the results relative to the Spatial FOD and the Temporal FOD analyses are reported below, in Tables 7 and 8 respectively, and only for the years 2015–2018. These changes do not greatly affect the Spatial FOD: the 2015 and 2018 rankings remain almost the same for the best performing areas. In particular, for 2018 it only emerges that Tete gets a worse ranking when asset and/or cooking fuel are dropped, whereas Cabo Delgado obtains a slightly better ranking. However, the maximum change in ranking is three positions and changes are in most cases equal to only one position.

**Table 7:** Spatial FOD multidimensional poverty comparisons, rankings of deprivation, 2015–18: Original and alternative specifications. *Source*: Authors’ computations

Area	Original specification		Alternative specification #1: Cooking fuel excluded		Alternative specification #2: Asset excluded		Alternative specification #3: Cooking fuel and asset excluded	
	Ranking 2015	Ranking 2018	Ranking 2015 (and change)	Ranking 2018 (and change)	Ranking 2015 (and change)	Ranking 2018 (and change)	Ranking 2015 (and change)	Ranking 2018 (and change)
Maputo City	1	1	1 (0)	1 (0)	1 (0)	1 (0)	1 (0)	1 (0)
Maputo Province	2	2	2 (0)	2 (0)	2 (0)	2 (0)	2 (0)	2 (0)
Urban	3	3	3 (0)	3 (0)	3 (0)	3 (0)	3 (0)	3 (0)
Gaza	4	4	4 (0)	4 (0)	4 (0)	4 (0)	4 (0)	4 (0)
Sofala	6	5	5 (− 1)	5 (0)	6 (0)	6 (+ 1)	6 (0)	5 (0)
National	5	6	6 (+ 1)	7 (+ 1)	5 (0)	5 (− 1)	5 (0)	6 (0)
Inhambane	7	7	7 (0)	6 (− 1)	7 (0)	7 (0)	7 (0)	7 (0)
Nampula	10	8	11 (+ 1)	8 (0)	8 (− 2)	8 (0)	8 (− 2)	8 (0)
Manica	9	9	9 (0)	9 (0)	10 (+ 1)	10 (+ 1)	11 (+ 2)	10 (+ 1)
Niassa	11	10	8 (− 3)	10 (0)	11 (0)	9 (− 1)	10 (− 1)	9 (− 1)
Tete	12	11	12 (0)	12 (+ 1)	12 (0)	14 (+ 3)	12 (0)	13 (+ 2)
Cabo Delgado	8	12	10 (+ 2)	11 (− 1)	9 (+ 1)	11 (− 1)	9 (+ 1)	11 (− 1)
Zambézia	14	13	13 (− 1)	13 (0)	14 (0)	13 (0)	13 (− 1)	12 (− 1)
Rural	13	14	14 (+ 1)	14 (0)	13 (0)	12 (-2)	14 (+ 1)	14 (0)

The changes in ranking compared to the original specification are included in parentheses for the alternative specifications

**Table 8:** Temporal net FOD multidimensional poverty comparisons, 2015–18: Original and alternative specifications. *Source*: Authors’ computations

Area	Original specification	Alternative specification #1: Cooking fuel excluded	Alternative specification #2: Asset excluded	Alternative specification #3: Cooking fuel and asset excluded
	2018 FOD 2015	2018 FOD 2015	2018 FOD 2015	2018 FOD 2015
National	0.03	0.03	0.73	0.74
Rural	0.04	0.04	0.65	0.67
Urban	0.11	0.11	0.17	0.17
Cabo Delgado	− 0.15			
Gaza	0.05	0.05	0.05	0.05
Inhambane	0.01	0.15	0.05	0.43
Manica	0.02	0.04	0.02	0.04
Maputo City				
Maputo Province	0.22	0.23	0.26	0.28
Nampula	0.09	0.22	0.12	0.37
Niassa				
Sofala	0.06	0.07	0.16	0.28
Tete				
Zambézia	0.03	0.04	0.16	0.26

Empty cells indicate that the comparison is indeterminate, which entails that the results provide no evidence of improvement for some year in some area/region. Net probabilities of temporal FOD are obtained via bootstrap

Conversely, with respect to the Temporal FOD, when we eliminate either the asset indicator or both the asset and the cooking fuel indicator this changes the picture. While dropping only the cooking fuel indicator leaves the results mostly unaltered, eliminating the asset indicator increases the probability of advancement over time for the entire country to 73%, for rural areas to 65% and for urban areas to 17%.^23^ The comparison over time for Cabo Delgado becomes undetermined instead of showing a negative probability and the probabilities of advancement are higher than in the original specification also for Inhambane, Maputo Province, Nampula, Sofala and Zambezia, even though the increase in the latter cases are not as pronounced as in the national and rural areas cases.

These last checks, we believe, on the one hand show that changing the specification in a multidimensional poverty evaluation can have important consequences in terms of rankings and especially in terms of estimated probabilities of advancement (Permanyer & Hussain, 2018). Nonetheless, they also—and perhaps more importantly—show that the asset indicator really seems to be one of the key variables shaping the evolution of well-being in Mozambique in the 2015–2018 period, as discussed in the conclusions. Therefore, the elimination of this indicator would hardly be justified in the present analysis, even though dropping it would depict a more favourable outcome in terms of probabilities of advancement for the whole country and for some of its regions.

Finally, we perform a robustness check including one indicator linked to education, i.e. the years of education of the woman interviewed. This is not an indicator that is listed among those used in the standard MPI or in the Global MPI, so we did not consider it in our main estimation set, but it may still provide an approximation of the education level in the household.

We present the results for this robustness check using only the MPI methodology. Depending on the weight allocated to the education indicator considered the level of multidimensional poverty changes. For example, if we follow the general MPI structure and allocate to the two available dimensions (education and living standards) equal weight, we end up with much lower overall deprivation levels. Nonetheless, the trends are confirmed and they reinforce the general results of the study (Table 9, panel a, and Fig. 8). That is, overall deprivation did not reduce much between 2015 and 2018.

**Table 9:** Poverty incidence, *H*, and MPI, *M*^*0*^, including one indicator linked to education, 2009–18. *Source*: Authors’ computations

	(a)				(b)			
	Equal weight allocated to the dimensions available (1/2 to education and 1/2 to living standards)				Equal weight allocated to the indicators available (1/7 to all indicators)			
	2009	2011	2015	2018	2009	2011	2015	2018
Original specification								
H	0.874	0.833	0.786	0.754	0.874	0.833	0.786	0.754
M^0^	0.732	0.699	0.630	0.605	0.732	0.699	0.630	0.605
Education indicator included								
H	0.665	0.664	0.581	0.545	0.873	0.830	0.790	0.752
M^0^	0.365	0.441	0.381	0.343	0.642	0.633	0.577	0.543

The poverty incidence, *H*, and MPI, *M*^*0*^, presented in panel (a) are obtained using the following weights: years of education of the woman interviewed (1/2), Living standards (1/2) (which entails that: Sanitation = 1/12, Drinking water = 1/12, Electricity = 1/12, Assets = 1/12, Housing = 1/12, Cooking fuel = 1/12). The poverty incidence, *H*, and MPI, *M*^*0*^, presented in panel (b) are obtained using the following weights: years of education of the woman interviewed = 1/7, Sanitation = 1/7, Drinking water = 1/7, Electricity = 1/7, Assets = 1/7, Housing = 1/7, Cooking fuel = 1/7

This conclusion becomes even more evident when all the indicators (i.e. the education indicator and the six living standards indicators) have the same weight (i.e., 1/7) (Table 9). In this case, the poverty incidence estimated for all years remains almost the same, the MPI being slightly lower in the case in which the education indicator is added to the analysis (Table 9, panel b).

## Footnotes

1 See Baez et al. (2018) and Salvucci and Santos (2020) for broader discussion and analyses on the relation between natural shocks and poverty in Mozambique.

2 That is up until the onset of the COVID-19 crisis.

3 The DHS guide to DHS statistics affirms that: *“The DHS Program was established by the United States Agency for International Development (USAID) in 1984. Originally designed as a follow-up to the World Fertility Survey (WFS) and the Contraceptive Prevalence Survey (CPS) projects, it has provided technical assistance to more than 350 surveys in over 90 countries, advancing global understanding of health and population trends in developing countries. […] The DHS Program has been implemented by the same team since its inception although the name of the organization has changed through acquisitions. It was first awarded in 1984 to Westinghouse Health Systems, which subsequently evolved into part of Macro Systems, ORC Macro, Macro International and is now part of ICF. The DHS Program is implemented by ICF and its partners Path, Avenir Health, Johns Hopkins Center for Communication Programs, Vysnova, Blue Raster, and Encompass. […] One of the key aims of The DHS Program is to collect data that are comparable across countries. To achieve this, standard model questionnaires have been developed.”* (DHS, 2021).

4 As the DHS guide to DHS statistics states: *“The Demographic and Health Survey was the original survey tool developed by The DHS Program in 1984. […] The DHS Program has evolved over the years to incorporate several other survey types: The DHS Program created the AIDS Indicator Survey (AIS) in 2003 to respond to the need for global monitoring of the HIV/AIDS epidemic. The AIS is a household based survey focusing on HIV and AIDS knowledge, attitudes, behavior, and prevalence. Demand for the AIS was not as high as had been anticipated, largely because the DHS was also designed to collect the same HIV data as a subset of the larger DHS and most countries requested a DHS rather than an AIS. Eleven AIS surveys were conducted in 2003–2015. In 2006, The DHS Program began implementing the Malaria Indicator Survey (MIS), also a household based survey, designed to collect data focused on internationally accepted malaria indicators. More than 30 MIS have been conducted since 2006. Though a DHS collects the same malaria indicators as an MIS, differences between the two surveys include the collection of malaria parasitemia and data collection during malaria high transmission season during an MIS, whereas a DHS typically collects data during the dry season.”* (DHS, 2021).

5 INS has been the responsible counterpart in Mozambique for all the DHS-related surveys. However, from 2018 the INS is no longer subordinate to MISAU, passing to be only overseen by it. This explains why the reference for the most recent survey is INS and ICF International (2019), whereas for previous surveys the suggested references only mention MISAU or include both MISAU and INS. With respect to the reference for the 2009 survey report, MISAU, INS, INE, & ICF Macro (2010), it mentions ICF Macro, but, as explained below, Macro has become part of ICF.

6 Consequently, results in the DHS/MIS 2018 report are presented taking as granted the representativeness at the different levels discussed. For example, it states that *“In Mozambique, 64% of households have access to an improved source of drinking water (Table 2.1.1). Eighty-nine percent of urban households and 53% of rural households have access to improved sources of drinking water. […] Apart from Maputo City (100%) and Maputo Province (96%), access to drinking water from improved sources is highest in Sofala (79%) and lowest in Cabo Delgado (44%) (Table 2.1.2)”* (INS & ICF International, 2019).

7 See also Alkire (2016) and Robeyns and Byskov (2021).

8 Alkire (2016) interprets “a person’s being deprived in at least a minimum set of widely valued achieved functionings as unfreedom, or capability poverty”.

9 For a more detailed and updated description of the indicator definitions, see Alkire et al. (2019a, 2019b).

10 In the multidimensional poverty analysis for Mozambique contained in DEEF (2016), both access to quality sanitation and to safe drinking water were considered as health correlates.

11 The variable measuring the time to drinking water facilities was not available in the 2009 survey. We therefore predicted the values for this variable using the 2011 DHS survey, regressing the time to water on a series of household and geographic characteristics. They include household head age and gender, household size, month of interview, province and urban/rural dummies, dummies for type of water source and type of roof, access to electricity, owning a car or a truck, owning a mobile phone, and owning a watch.

12 In the original formulation by the Oxford Poverty and Human Development Initiative (OPHI), the household is classified as deprived with respect to assets if it does not own more than one of these assets: radio, television, telephone, computer, animal cart, bicycle, motorbike, or refrigerator, and does not own a car or truck. However, in the surveys considered, the information on possession of a computer is not consistently available, so we excluded this item from the analysis. The impact on the results will be minimal given the very low percentage of Mozambicans owning a computer, as recorded in other household surveys.

13 We also present in Sect. 4.3.1 and in the “Appendix” results with different cut-offs for robustness.

14 The global MPI 2019 uses 33.3% (or 1/3 of the weighted welfare indicators) as cut-off, but in Mozambique, this cut-off appears very low given the widespread levels of deprivation. Multidimensional poverty results obtained using a cut-off of 33.3% have been computed and are in Fig. 6.

15 For a description of the method and application to the Mozambican and other country cases, see Arndt et al. (2012, 2016a, 2016b), and Arndt and Tarp (2017).

16 A Wald test of means was performed, which allows to take care of the survey settings (e.g. see UCLA, 2020).

17 Population data are obtained from the World Population Prospects 2019 (see United Nations, 2019).

18 This is also the procedure followed by OPHI in the global MPI (for details, see the ‘Data tables 2019’ in OPHI, 2019).

19 An additional temporal FOD analysis was performed including the three regions of Mozambique, north, centre, and south. In this case, we estimated a probability of advancement for the centre of only 1%; we obtain no evidence of advancement for the north, and a sizeable positive probability of advancement for the south, 79%.

20 In addition to that, Mahrt et al. (2020) report that when Mozambican children’s wellbeing is compared with that of children in other countries in the region, their MPI substantially exceeds that in neighboring countries.

21 More details on this are added in the conclusions when discussing further steps.

22 Only the complete DHS databases contain all the information regarding the health, education and living standards indicators needed to compute the country-specific MPIs that feed the Global MPI.
